# Meeting Report: Batch-to-Batch Variability in Estrogenic Activity in Commercial Animal Diets—Importance and Approaches for Laboratory Animal Research

**DOI:** 10.1289/ehp.10524

**Published:** 2007-12-05

**Authors:** Jerrold J. Heindel, Frederick S. vom Saal

**Affiliations:** 1 Division of Extramural Research and Training, National Institute of Environmental Health Sciences, National Institutes of Health, Department of Health and Human Services, Research Triangle Park, North Carolina, USA; 2 Division of Biological Sciences, University of Missouri, Columbia Missouri, USA

**Keywords:** animal diets, batch-to-batch variability, bioassay, estrogenic contaminants, isoflavones, phytoestrogens

## Abstract

We report information from two workshops sponsored by the National Institutes of Health that were held to *a*) assess whether dietary estrogens could significantly impact end points in experimental animals, and *b*) involve program participants and feed manufacturers to address the problems associated with measuring and eliminating batch-to-batch variability in rodent diets that may lead to conflicting findings in animal experiments within and between laboratories. Data were presented at the workshops showing that there is significant batch-to-batch variability in estrogenic content of commercial animal diets, and that this variability results in differences in experimental outcomes. A combination of methods were proposed to determine levels of total estrogenic activity and levels of specific estrogenic constituents in soy-containing, casein-containing, and other soy-free rodent diets. Workshop participants recommended that researchers pay greater attention to the type of diet being used in animal studies and choose a diet whose estrogenic activity (or lack thereof) is appropriate for the experimental model and end points of interest. Information about levels of specific phytoestrogens, as well as estrogenic activity caused by other contaminants and measured by bioassay, should be disclosed in scientific publications. This will require laboratory animal diet manufacturers to provide investigators with information regarding the phytoestrogen content and other estrogenic compounds in commercial diets used in animal research.

## Overview of the Meetings

In this report we will discuss implications for researchers of variability in estrogenic activity in animal feed and provide examples of a number of end points that are sensitive to estrogenic components in rodent diets. Importantly, we will report how research is impacted by variability in estrogenic components in soy-based and casein-based diets. We also describe control procedures to address the issue of batch-to-batch variability in animal diets. A primary concern expressed during these two workshops sponsored by the National Institutes of Health (NIH) was a need to increase awareness within the scientific and laboratory animal medicine community about the variability in estrogenic activity in diets associated with different components used in diets and the potential impact on a wide range of end points.

The primary objective of the first workshop was to review the literature to assess whether dietary estrogens could significantly affect hormonal and other end points in experimental animals. Based on the consensus that there were significant effects, a second workshop was held to develop strategies to reduce the problems caused by batch-to-batch variation in estrogenic compounds in animal diets. Meeting participants agreed that it was critical to inform scientists, laboratory animal medicine personnel, animal caretakers, scientific societies, and funding agencies about these issues. Participants also developed a plan that provides a solution for confirming the level of estrogenic activity in each diet in order to address the problem of undefined batch-to-batch variability in estrogenic activity in diets.

The following critical issues were discussed at the two workshops:

- Even though the protein content of soy-containing animal diets is constant, the phytoestrogen content can vary 2- to 5-fold between different batches of the same commercial rodent diet. Other estrogenic contaminants also vary from batch to batch.
- Phytoestrogens in soy-based animal diets are biologically active and affect experimental results, including masking the effect of positive control estrogens. There are species and strain differences in the response to variation in dietary phytoestrogens.
- Research findings support the need for laboratory animal diet manufacturers/vendors to confirm content and activity of estrogenic compounds in at least some of their rodent diets and make this information available to investigators. These assays should ideally be done on a batch-to-batch basis for both soy-containing and for soy-free diets so that researchers will be able to use batches with similar estrogenic content.
- These findings also emphasize the importance of the diet in rodent studies, because uncontrolled variability in estrogenic activity in feed can interfere with replication of results within and between laboratories. There is thus a need to educate investigators about these issues.

Findings presented at the workshops provided evidence that some batches of diets with no measurable isoflavones (and thus assumed by many scientists to be estrogen-free) contained other substances with estrogenic activity, such as zearalenone, a nonsteroidal estrogen produced by mold (Katzenellenbogen et al. 1979), and other unidentified estrogenic compounds. These findings provide a rationale for the recommendation to use bioassays to complement chemical analysis in assessing the total estrogenic activity of rodent diets.

## Types of Feed and Sources of Variability in Estrogenic Activity in Commercial Animal Feed

### Types of laboratory animal diets

Three major types of commercial diets produced for laboratory rodents were discussed at the workshops: *a*) natural ingredient diets, which are formulated with agricultural products and byproducts such as whole grains (e.g., ground wheat, ground corn, ground oats), mill byproducts (e.g., wheat bran, wheat middlings, corn gluten meal), and high-protein meals (e.g., soybean meal, alfalfa meal, fishmeal), and contain added mineral and vitamins; *b*) purified rodent diets, which are formulated with refined ingredients such as casein or soy protein isolate, sugar, starch, vegetable oil, and cellulose (Reeves 1997); and *c*) chemically defined diets, which are formulated using chemically pure compounds such as amino acids, sugars, triglycerides, fatty acids, and inorganic salts (Knapka 1985). All natural ingredient laboratory animal diets can be classified as being either open- or closed-formula diets. For open-formula diets, the quantitative ingredients are published and open to the public. In contrast, for closed formula diets, only the ingredients are listed, but the precise concentration of components is unknown or proprietary.

### Impact on phenotype of phytoestrogen levels in soy-containing diets

Various plant species naturally produce hormonally active compounds. These include, but are not limited to, the isoflavones from soy, coumestrol (a coumestan found in alfalfa), lignans from flaxseed, and some fungal mycotoxins, such as zearalenone, that contaminate plant products. However, the primary known source of active estrogenic chemicals present in most rodent diets is isoflavones. The two main isoflavone conjugates, genistin and daidzin, are not estrogenic, but they are hydrolyzed in the intestinal tract to form the aglycones, genistein and daidzein, which have estrogenic activity (Setchell et al. 2002). In addition, equol, a metabolite of daidzein, is formed by bacteria in all rodents fed soy-containing diets and in some humans consuming soy foods (Setchell and Cole 2006). Equol has estrogenic activity and is also an antiandrogen that antagonizes the actions of dihydrotestosterone *in vitro* (Lund et al. 2004).

A survey of available diets revealed that most of the commonly used commercial rodent diets are formulated with significant levels of soy protein, and plasma and urinary isoflavone concentrations correlate well with dietary isoflavone intake in mice and rats (Brown and Setchell 2001; Thigpen et al. 2003), similar to findings from humans and other animals (Setchell et al. 1987a, 1997). Setchell and Cole (2003) described a high variability in the isoflavone content of purified soy protein isolates and soy foods sold to humans, highlighting the difficulty that manufacturers of the raw ingredients have in controlling for isoflavone levels in soy-protein products. A large variability in isoflavone content is found in different species of soybeans; this is caused by genetic and environmental factors, use of pesticides, and also changes occurring with processing and storage (Hoeck et al. 2000; Hou and Chang 2002; Tsukamoto et al. 1995). Natural fluctuations in sources of soy protein will result in batch-to-batch variation in total isoflavone content of the diet, even if the concentration of soy protein is kept constant. Thus, variability in estrogenicity of soy-based diets is to be expected. There was consensus at the workshops that laboratory diet manufacturers basically have no means to control the level of isoflavones without varying the soy protein content of the diet. Because of the difficulty in maintaining a constant soy-protein content, rodent diets will vary dramatically in estrogenic potency from batch to batch and among different manufacturer’s diets.

Both endogenous and exogenous estrogens have a very wide range of effects in both males and females. These effects are mediated by both the classical nuclear estrogen receptors (ER-α and ER-β), which are ligand-activated transcription factors, and the more recently discovered ERs associated with the cell membrane, which act via second-messenger systems to cause rapid changes in cell function (reviewed by Welshons et al. 2006). The soy isoflavone genistein and equol, the major circulating isoflavone metabolites in rodents fed soy diets, have higher affinity for the nuclear ER-β than the classical ER-α. These are then, by definition, selective ER modulators (SERMs).

At the workshops, numerous examples were presented showing the relationship between levels of isoflavones in feed and a variety of traits in rats and mice, such as the timing of opening of the vaginal canal, an estrogen-sensitive biomarker of sexual maturation in mice (Thigpen et al. 2003, 2004b); the timing of embryo implantation in rats (Wang et al. 2005); and the incidence and timing of onset of spontaneous vulvar carcinomas in mice (Thigpen et al. 2001). Thigpen et al. (2004b) suggested that diets essentially lacking in known sources of phytoestrogens should be fed to females when they are being examined after weaning and before puberty, when endogenous levels of estradiol are very low.

In contrast, other workshop participants suggested that the use of soy-free diets could be problematic, depending on the life stage, experimental design, animal model, and end points assessed. For example, a paradoxical finding is that the absence of estrogenic activity in the feed during pregnancy resulted in the “fetal estrogenization syndrome” and obesity during later adulthood in CD-1 mice (Ruhlen et al. 2008). Additional research has shown marked differences in metabolism in CD-1 mice fed estrogen-free diets compared with those fed diets containing phytoestrogens beginning at conception, with the absence of soy isoflavones resulting in an increase in body weight, a decrease in glucose tolerance, decreased metabolic rate and activity level, and other metabolic changes associated with obesity (Cederroth et al. 2007). These findings illustrate the potential for a wide range of impacts on phenotype of eliminating phytoestrogens and other estrogenic contaminants commonly found in commercial diets. Investigators need to pay particular attention to the potential effect of diet on all end points, not just end points such as vaginal opening that are used as common bioassays for estrogen exposure (Cooke and Naaz 2004; Jensen and Ritskes-Hoitinga 2007).

### Strain differences in the response to isoflavones in feed

Significant differences have been found in the responsiveness of different species and strains of animals to dietary phytoestrogens (Thigpen et al. 2004a). For example, the CD-1 mouse and F344 rat are both extremely sensitive to variation in the levels of isoflavones in phytoestrogen-containing diets, whereas the Sprague-Dawley rat is significantly less sensitive, as measured by the effect of different diets on the timing of vaginal opening (Thigpen et al. 2007). This finding is consistent with several reports showing the Sprague-Dawley rat to be less responsive than the F344 rat to other xenoestrogens (vom Saal and Hughes 2005).

### Life stage

Workshop participants noted that it is critical to consider life stage when examining the effect of phytoestrogens on phenotype. It is uncommon for commercial vendors of laboratory animals to inform investigators or laboratory animal care personnel of the specific diet(s) used during breeding and/or maintenance of animals before delivery to the research facility. Such information is highly relevant because, during critical periods in development, components of the diet can result in programming of genes, resulting in gene silencing or gene activation (Anway and Skinner 2006; Dolinoy et al. 2007).

## Batch-to-Batch Variability in Estrogenic Activity in Soy-Containing Diets

Central to the focus of these workshops was the issue of variability in research results associated with batch-to-batch variability in the phytoestrogen content of rodent diets that contain soy protein. The ratio of soy isoflavones to total protein in purified soy-protein isolates used as raw material to formulate soy food can vary by 3- to 5-fold; this variability is exclusively due to the variability in isoflavone content, because total protein content generally shows little (~ 3%) variation (Setchell and Cole, 2003; Thigpen et al. 1999, 2003). This variability is exacerbated when the amount of soy-protein ingredients also varies from batch to batch, as is the case in closed-formula diets that are changed to maintain constant nutrition.

In several studies of prepubertal female rats and mice, a failure to reproduce uterine weight changes and timing of vaginal opening was shown to be the result of batch-to-batch variability in the phytoestrogen content of the diet. This disruption of research due to batch-to-batch variability in feed occurred in different experiments that involved diets purchased from two different feed manufacturers, indicating that this is not a problem associated with any specific manufacturer, but is, instead, due to variability in the isoflavone content of soy (Boettger-Tong et al. 1998; Thigpen et al. 2003, 2004a, 2004b). For example, Thigpen et al. (2003) reported that a batch of Purina 5002 diet containing a high background level of phytoestrogens disrupted the ability to use the vaginal opening bioassay to detect the well-characterized puberty-accelerating action of the potent synthetic estrogen diethylstilbestrol (DES), whereas DES effects were observed with another batch of Purina 5002 that had much lower phytoestrogen levels.

## Controlling versus Eliminating Batch-to-Batch Variation in Estrogenic Activity in Animal Diets

The most logical and practical approach for eliminating variability of estrogenic activity in rodent diets would be to eliminate dietary ingredients that contain known estrogenic compounds (Thigpen et al. 1998, 1999, 2004b). Many researchers have used soy-free diets and suggested that these diets should be used for studies of estrogenic chemicals (Casanova et al. 1999; Kanno et al. 2002; Stroheker et al. 2003; You et al. 2002). Indeed, use of non-soy/alfalfa-based diets would permit a physiology and phenotype to be established in the absence of phytoestrogens. However, Thigpen et al. (2004b) cautioned that the diet selected for any given study should depend on the specific objective(s) of the study, and that the diet selected should reduce study variability, not increase study variability.

The findings presented at the workshop clearly demonstrated that animals fed soy/alfalfa-free diets might not be comparable to historical data generated in animals fed diets high in phytoestrogens; of particular concern was that the absence of soy in the feed led to obesity in CD-1 mice (Cederroth et al. 2007; Ruhlen et al. 2008). Whether changing the type of protein in the diet from soy to casein results in a different but normal phenotype or a different but abnormal phenotype is still a question that will require additional research in different animal models, but an impact on phenotype should be expected. The source of the protein may not be as important as the estrogenic activity of the diet, including the unique profile of estrogenic contaminants that contribute to the total estrogenic activity. The use of soy- or non-soy–containing diets is an issue that researchers should carefully consider as part of their experimental design; at the workshops there was agreement that there is no one optimum diet for conducting all types of research.

An important consideration regarding the choice of animal feed is that batch-to-batch variability in estrogenicity has also been observed in soy-free rodent diets that contain casein as a protein source. Researchers should be aware that a soy-free diet cannot automatically be assumed to be an estrogen-free diet, because there are many potential sources of estrogenic contaminants in the various ingredients used in commercial soy-free feeds (vom Saal et al. 2004).

## Measuring and Controlling Batch-to-Batch Variability in Estrogenic Components of Commercial Rodent Diets

Workshop participants proposed a paradigm combining two approaches to provide information on the batch-to-batch variability in estrogenic activity in commercial diets: chemical analysis and bioassay.

### Chemical analysis for known estrogenic compounds

For diets that contain components such as soy, chemical analysis should be conducted for specific chemical classes that are sources of variability in estrogenic activity. These include the isoflavones genistein and daidzein, glycitein, formononetin, and biochanin A as well as their respective glucoside conjugates. This can be accomplished using techniques described previously by many groups (Coward et al. 1993; Setchell and Cole 2003; Setchell et al. 1987b). Because the isoflavone glucosides do not possess estrogenic activity *in vitro* unless they are hydrolyzed in the gastrointestinal tract to release the bioavailable aglycones (genistein, daidzein, and glycitein), hydrolysis of the diet should ideally precede measurement by HPLC, with ultraviolet, electrochemical, or mass spectrometry detection (Setchell and Cole 2003; Setchell et al. 1987b). The approach of measuring total isoflavone concentration (as opposed to attempting to measure the low proportion that is not glycosylated) greatly simplifies the method. Commercial methods are available for the detection of the mycotoxin zearalenone; if alfalfa is included in the diet, coumestans can be determined by HPLC (Cassidy et al. 2000). The advantage of this analytical approach is that it will absolutely detect specific estrogenic compounds and identify their levels. The disadvantage is that it is a biased assay that will not detect other components of the diet that may also have estrogenic activity.

### In vitro *screening bioassays for total estrogenic activity*

To complement the chemical analyses, a combination of two *in vitro* bio-assay methods can be used to screen for estrogenic activity in rodent diets: one bioassay to determine if there is estrogenic activity in the diet, and a follow-up bioassay that could determine the profile of the contaminants. These assays have the advantage that they are unbiased and permit the detection of multiple unknown components that may have estrogenic activity.

An example of a bioassay that uses human MCF-7 breast cancer cells was described by Welshons et al. (1990). There are other bio-assays for estrogenic activity (Jefferson et al. 2002; Thigpen et al. 2001), but they should be evaluated to determine whether they have the high sensitivity, specificity, and high-throughput capability of the MCF-7 cell proliferation bioassay. Use of such assays would allow a more expansive assessment of the presence of potential estrogenic chemicals in the diets. A drawback of this approach is that these types of bioassays are unable to establish the identity of the estrogenic chemicals; nonspecific interferences can potentially lead to false positives unless the results are supported by a chemical identity of the estrogenic compound. These assays would also have limited value in detecting conjugated forms of estrogens unless the diet extracts were subjected to prior hydrolysis. Nevertheless, this approach could be helpful in initial screening of diets using high-throughput techniques and would alert manufacturers to potential estrogenic activity of specific batches of a diet. These assays are also able to identify compounds with antiestrogenic activity by administering a stimulating dose of estradiol and then determining whether the response to estradiol is reduced.

Diets assumed to have few or no components with estrogenic activity have been found to contain estrogenic contaminants; therefore, this bioassay approach is needed even if manufacturers or researchers assume that the diet is free of or low in estrogenic activity. Thus, workshop participants agreed that although purified diets and non-soy/alfalfa–containing diets would not need to be subjected to chemical analysis for compounds that are not components of the diet, they should still be tested for total estrogenic activity by a sensitive bioassay.

### Creating a profile of the estrogenic activity in feed by HPLC and bioassay

For batches of feed that test positive in a bioassay screen, it was proposed that an additional assay should be applied to create a profile of the estrogenic substances. This second-tier assay would potentially facilitate identifying the component(s) of the feed that contributed the contaminants. The objective is that once the source of the contaminant(s) is identified, removal can be addressed. This second-tier bioassay is referred to as generating a fingerprint of the extracted estrogenic compounds (for examples, see Grady et al. 1991; Howdeshell et al. 2003). The fingerprint would be determined by extracting the estrogenic compounds, separating them by HPLC, and adding the fractions to cultures containing estrogen-responsive cells, such as MCF-7 cells. This creates a profile or fingerprint of fractions that stimulates estrogen-dependent cell proliferation. This follow-up fingerprint bioassay would only be conducted if estrogenic activity had been detected in the diet in the first bioassay for total estrogenic activity. An important advantage of this second-tier bioassay is that it would provide information as to whether the total estrogenic activity in different batches of a diet is due to the same profile of estrogenic compounds or to a different profile. In the latter case, there would be concern that specific compounds present in one batch but not another could act as SERMs with unique effects on phenotype.

Meeting participants agreed that, taken together, these two bioassays would provide a method for monitoring total estrogenic activity in a diet and also provide additional information concerning variability in the components used in preparing different batches of a diet. One limitation of this approach is that any estrogenic compound that is conjugated or activated by *in vivo* metabolism would not be detected. Therefore, complementary approaches using both bioassay and chemical assays for detecting and measuring estrogenic substances in diets is advantageous, provided they are technically feasible and cost-effective.

## Conclusions and Recommendations

Participants of these two workshops concluded that investigators would benefit from the availability of commercial animal diets for which manufacturers have identified the components responsible for estrogenic activity and applied appropriate manufacturing quality control procedures to assess the variability between batches of the diets. Documentation of the estrogenic activity in diets by chemical analysis coupled with a bioassay to identify potential batch-to-batch variability in estrogenic composition can effectively meet this need.

Specifically, feed manufacturers should *a*) make available animal diets for which the level of estrogenic activity is identified based on bioassay and chemical analysis; and *b*) make available diets in which the estrogenic activity falls within specified ranges, including diets devoid of estrogen activity.

The following research needs were identified on the basis of data gaps noted at the meeting:

- What end points are most sensitive or are insensitive to the effects of dietary phytoestrogens and other estrogenic contaminants?
- What nonclassical estrogenic effects occur because of variability in estrogenic chemicals in feed?
- What are the most sensitive life stages for effects of phytoestrogens and other estrogenic contaminants on end points?
- What is the optimal range of phytoestrogens and total estrogenic activity in diets with regard to optimal health and responsiveness to experimental treatments?

In conjunction with the manufacturer-based approaches described above, participants discussed approaches that can be implemented by the scientific community, as well as regulatory agencies that oversee the conduct of research on drugs and chemicals:

- Details of diet composition, mill dates, and the estrogenic content should by adequately documented in publications, which may require the use of open-formula diets. This emphasizes the need for standardized assurance methods.
- Researchers should be aware of implications of estrogenic activity in the diet, and be willing to modify the diet as appropriate for the specific experimental protocol and end points.
- Researchers should be aware of species, strain, and life stage differences in effects of phytoestrogen content and other estrogenic chemicals in the diet being used.
- Positive controls should be used in all experiments. Researchers should not rely on historical control data because estrogenic contaminants in prior batches of animal diets, even those assumed to be estrogen-free, are likely to vary from current batches.
- Samples of diets should be held for future analysis of potential contaminants.

Most biomedical researchers are generally unaware of the type of diets used in a central animal facility, and information about diet is often lacking in published reports, despite the importance of the diet, which has been previously highlighted in the scientific literature (Brown and Setchell 2001; Jensen and Ritskes-Hoitinga, 2007; Thigpen et al. 2003, 2004a, 2004b). The workshop participants felt that it would be helpful for journals to request a detailed description of diets, mill dates, and estrogenic activity, including a description of how estrogenic activity was measured. Scientific societies could also facilitate awareness of this problem through symposia and workshops at meetings and in journal articles. In addition, there is concern about the potential for animal research to be impacted by variability in other contaminants, such as methylmercury (Weiss et al. 2005) and polychlorinated biphenyls (Environmental Working Group 2003), which have been proposed to relate to the use of contaminated fish meal in some diets. Thus, although our focus has been on controlling variability in estrogenic compounds in rodent diets, we hope that other potential sources of uncontrolled variability in research results associated with components of the diet will also be addressed by laboratory animal diet manufacturers as new findings are published.

Although the workshops focused on animal studies, it seems clear that variation in diet components may also affect clinical trials; also, that quality of research would be improved if more emphasis is placed on dietary components and how they may alter experimental results at all levels. Participants felt it may be worthwhile having a trans-NIH workshop to further probe this issue and its effects on both animal and human experimental protocols.

We are optimistic about the utility and manufacturing feasibility of the approaches presented because workshop participants included investigators undertaking relevant research, government scientists, and representatives from animal feed manufacturers.
